# Efficacy and safety of regorafenib plus biweekly trifluridine/tipiracil for refractory metastatic colorectal cancer: a multicenter single-arm phase II trial

**DOI:** 10.1093/oncolo/oyaf129

**Published:** 2025-06-17

**Authors:** Xiangling Wang, Zhen Li, Dan Sha, Haipeng Ren, Cuihua Yi, Shuguang Li, Peng Wang, Yunxia Chu, Changlun Li, Guanglian Shan, Jian Wang, Xiaorong Yang, Jing Hao

**Affiliations:** Department of Medical Oncology, Qilu Hospital of Shandong University, Jinan 250012, People’s Republic of China; Department of Medical Oncology, Linyi Cancer Hospital, Linyi, People’s Republic of China; Department of Minimally Invasive Comprehensive Treatment of Cancer, Shandong Provincial Hospital Affiliated to Shandong First Medical University, Jinan 250012, People’s Republic of China; Department of Medical Oncology, Weifang People’s Hospital, Weifang, People’s Republic of China; Department of Medical Oncology, Qilu Hospital of Shandong University, Jinan 250012, People’s Republic of China; Department of Medical Oncology, Qilu Hospital of Shandong University, Jinan 250012, People’s Republic of China; Department of Oncology, Weifang Yidu People’s Hospital, Weifang, People’s Republic of China; Department of Medical Oncology, Qilu Hospital of Shandong University, Jinan 250012, People’s Republic of China; Department of Oncology, Affiliated Hospital of Jining Medical University, Jining, People’s Republic of China; Department of Medical Oncology, Xintai People’s Hospital, Taian, People’s Republic of China; Department of Medical Oncology, Qilu Hospital of Shandong University, Jinan 250012, People’s Republic of China; Department of Medical Oncology, Qilu Hospital of Shandong University, Jinan 250012, People’s Republic of China; Department of Medical Oncology, Qilu Hospital of Shandong University, Jinan 250012, People’s Republic of China

**Keywords:** regorafenib, trifluridine/tipiracil, refractory colorectal cancer

## Abstract

**Background:**

Both regorafenib and trifluridine/tipiracil (TAS-102) monotherapies have shown significant but limited survival benefits in metastatic colorectal cancer (mCRC) cases who progress after standard treatments. This study aimed to evaluate the efficacy and safety of regorafenib plus biweekly TAS-102 in refractory mCRC.

**Methods:**

In this single-arm multicenter phase II trial (ChiCTR2300071752), eligible patients received regorafenib at 120 mg/day for 21 days in a 4-week cycle or were treated with a dose-escalation strategy (80 mg/day, followed by weekly increase of 40 mg to 120 mg/day). TAS-102 was administered biweekly (30 mg/m^2^ bid on days 1-5). The primary endpoint was progression-free survival (PFS). The secondary endpoints included safety, response rate (ORR), disease control rate (DCR), and overall survival (OS).

**Results:**

Between March 1, 2022 and December 1, 2023, 28 patients were enrolled. Totally 24 patients had at least one response evaluation. Median PFS and OS were 4.9 months (95% CI, 4.2-5.6) and 15.4 months (95% CI, 11.1-19.7). The ORR was 8.3% and the DCR was 83.3%. Grade 3 or 4 treatment-related adverse events occurred in 21.4% of patients, including hypertension (7.1%), neutropenia (7.1%), thrombocytopenia (3.6%), and hoarseness (3.6%).

**Conclusions:**

Regorafenib plus biweekly TAS-102 showed promising benefits in refractory mCRC cases, and adverse events were generally tolerable and manageable.

**Discussion:**

(ClinicalTrials.gov Identifier: ChiCTR2300071752. IRB Approved: KYLL-202203-026-1.)

**Lessons learned**

- Regorafenib in combination with biweekly trifluridine/tipiracil provided increased PFS (4.9 months) and OS (15.4 months) compared with historic monotherapy in refractory CRC.
- Regorafenib plus biweekly trifluridine/tipiracil demonstrated a manageable safety profile.

Metastatic colorectal cancer (mCRC) remains a major cause of cancer-related death globally.^1^ Although initial systemic therapies, primarily fluorouracil-based chemotherapy combined with oxaliplatin or irinotecan and augmented with bevacizumab or EGFR antibodies, demonstrate notable benefits, most patients with microsatellite stable (MSS) eventually succumb to disease progression, with limited options in subsequent treatment lines.^2^ Currently, three drugs are approved for refractory mCRC cases, that is, regorafenib, trifluridine/tipiracil (TAS-102), and fruquintinib, with modestly extended progression-free survival (PFS, 1.9–3.7 months) and overall survival (OS, 6.4–7.4 months).^3-8^ Nevertheless, optimal selection and sequencing of these drugs, administered either sequentially or in combination, remain undetermined.^9-11^ The aggressive chemo-refractory disease nature and rapid patient performance status decline may preclude some cases from receiving all available treatments in clinical practice.^12^

Addressing these challenges in later-line settings, chemotherapy combined with an anti-angiogenic agent represents a promising approach. The SUNLIGHT trial^13^ demonstrated superior survival benefit with a combination of FTD-TPI and bevacizumab versus FTD-TPI alone, highlighting the importance of sustained angiogenesis inhibition and a combination strategy. However, the survival advantage of adding bevacizumab in mCRC cases with prior bevacizumab in both first- and second-lines (HR = 0.76, 95% CI, 0.52-1.1) in the SUNLIGHT study requires further evaluation.^14^ Since regorafenib inhibits various angiogenic and growth receptor tyrosine kinases and overcomes resistance to bevacizumab,^15^ substituting bevacizumab with regorafenib plus TAS-102 has emerged as another approach. The phase I REMETY study set the stage for this approach, confirming the feasibility and safety of a regorafenib and TAS-102 regimen on a conventional 4-week cycle, yielding a PFS of 3.8 months and an OS of 11.1 months.^16^

This study examined whether regorafenib in combination with biweekly TAS-102 for refractory mCRC is safe and beneficial. The primary endpoint was met, with median PFS and OS of 4.9 months (95% CI, 4.2-5.6) and 15.4 months (95% CI, 11.1-19.7) respectively, as depicted in Figure 2. The study also showed a manageable safety profile, with 21.4% grade 3 or 4 treatment-related adverse events.

**Figure 1.**
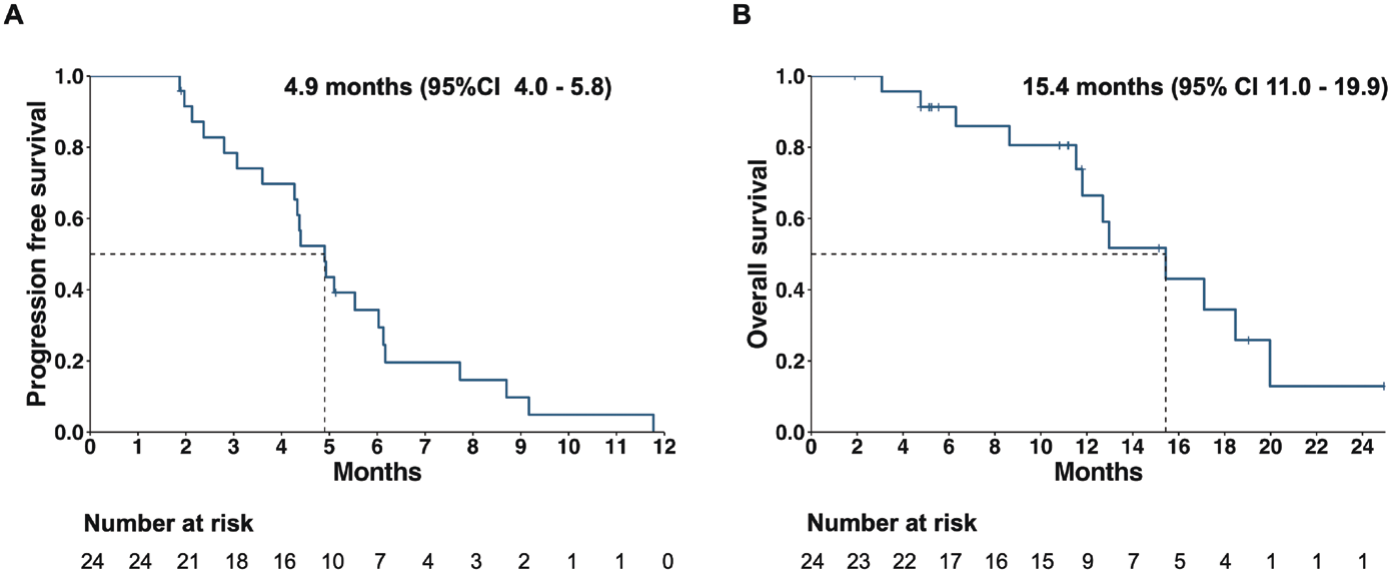
Consort diagram: study flowchart for safety and efficacy analyses.

**Table:** 

Trial information	
Disease	Colorectal cancer--CRC
Stage of disease/treatment	Refractory metastatic
Prior therapy	Prior treatment with 5-FU, oxaliplatin, irinotecan, anti-VEGF antibody and anti-EGFR antibody for wild type RAS, no prior TAS-102 or antiangiogenic tyrosine kinase inhibitor
Type of study	Open-label, single-arm, multi-center, phase II
Primary endpoint	Progression-free survival
Secondary endpoints	Safety, overall response rate, disease control rate, overall survival

Additional details of endpoints or study design

REGTAS was a single-arm, multicenter, phase II trial conducted in 7 hospitals from Shandong province, China. This study protocol was approved by Ethics Committee of Qilu Hospital of Shandong University and registered in the Chinese Clinical Trial Registry as ChiCTR2300071752.

## Patients

Eligible patients were aged 18 years or older with histologically confirmed microsatellite stable unresectable metastatic colorectal cancer, had a WHO performance status of 0 or 1, a life expectancy of at least 3 months, and had measurable lesions according to Response Evaluation Criteria in Solid Tumors (RECIST) version 1.1. Previous treatment must have included a fluoropyrimidine, irinotecan, oxaliplatin, bevacizumab, and/or an anti-EGFR antibody (in patients with RAS wild-type tumors). Eligibility requires knowledge of RAS status. The main exclusion criteria were: previously treated with either TAS-102 or antiangiogenic tyrosine kinase inhibitor (eg, regorafenib or fruquintinib); unstable cardiac disease; a history of arterial or venous thrombotic or embolic events within 6 months of informed consent.

## Study schedule and intervention

All eligible patients were administered regorafenib plus TAS-102. Regorafenib was administered once daily in a 120 mg or starting dose of 80 mg with weekly escalation, per 40 mg increment to 120 mg for 3 weeks on, 1 week off; TAS-102 was administered at 30 mg/m^2^ twice daily on days 1 through 5 every 2 weeks. Treatment continued until disease progression, death, unacceptable toxicity, consent withdrawal, or investigator discretion. If dose reduction was needed, TAS-102 was reduced in decrements of 5 mg/m^2^ until reaching a minimum dose of 40 mg/m^2^ daily, and regorafenib was reduced to 80 mg. In case of regorafenib delay, TAS-102 was permitted to be administered as planned, and vice versa. If patients had unacceptable toxicities related to either drug, monotherapy with the other one could be continued.

## Endpoints and assessments

The primary study endpoint was progression-free survival (PFS), and secondary endpoints included safety, response rate (ORR), disease control rate (DCR), and overall survival (OS). PFS was defined as the period from the time of enrolment to first disease progression or death from any cause, whichever occurs first. ORR was defined as the proportion of patients achieving a complete response (CR) or partial response (PR). DCR was the proportion of patients with CR or PR plus stable disease (SD). Radiologic assessments were performed by investigators at baseline and every 8 weeks (±1 week) until progression according to Response Evaluation Criteria in Solid Tumors (RECIST) version 1.1. Vital status was to be updated every 12 weeks until death or trial end. Data on adverse events and abnormal laboratory findings were collected regularly throughout the treatment period and for 30 days post-treatment. Adverse events were graded per the National Cancer Institute Common Terminology Criteria for Adverse Events, version 5.0. For patients administered only one cycle of treatment with dose interruption for more than 4 weeks due to COVID-19 pandemic, only side effects were assessed.

## Statistical analysis

The clinical benefit of TAS-102 monotherapy in refractory mCRC had been established from TERRA and RECOURSE trials, with a median PFS of 2.0 months, and data from phase I REMETY trial indicated this could increase to 3.8 months in patients when administered with regorafenib. If the actual median PFS for combination therapy is increased from historic upper limit of 2.5 months of TAS-102 monotherapy to 4 months, with a two-sided significance level of 0.05, a power of 90%, and a 20% dropout rate in COVID-19 pandemic period, a sample size of 24 patients was determined as necessary. All enrolled patients who initiated the study treatment were included in the safety set. Efficacy analyses were restricted to patients who had completed at least two treatment cycles and completed at least one response evaluation. The Clopper-Pearson method was used to calculate the 95% confidence intervals for ORR and DCR. PFS and OS curves were obtained by the Kaplan-Meier method, and data were reported with two-sided 95% confidence intervals (CI). Statistical significance was set at *P* < .05. Exploratory post-hoc subgroup analyses of PFS and OS were performed. All analyses were performed using SPSS software (version 26.0) and R version 4.3.2.

**Table:** 

Drug information	
**Generic/Working name**	Regorafenib / TAS-102
**Company name**	Bayer Phamaceutical Co./ Qilu Phamaceutical Co.
**Drug type**	Tyrosine kinase inhibitor / Cytotoxic agent
Drug class	Antiangiogenic / Chemotherapy
Dose	Regorafenib, 120 mg/day for 21 days in a 4-week cycle, or using a dose-escalation strategy (80 mg/day, followed by weekly increase of 40 mg to 120 mg/day). /TAS-102, 30 mg/m^2^ twice daily on days 1-5, biweekly.
**Unit**	Milligrams
**Route**	Oral
Schedule of administration	Q4W/Q2W

**Table:** 

Patient characteristics	
**Number of patients, male**	14
**Number of patients, female**	14
**Stage**	IV
**Median age (range)**	58 (33-73) years
**Number of prior systemic therapies**	2 lines: 25 ≥3 lines: 3
**Performance status: ECOG**	0: 11 1: 17
**Primary tumor location**	Left colon: 7 Right colon: 6 Rectum:15
**Duration of metastatic disease**	≤18 m: 17 >18 m: 11
**Genotype**	RAS mutations: 15 BRAF mutations: 2
**Previous treatment agents**	5-Fluorouracil: 28 Oxaliplatin: 28 Irinotecan: 28 Bevacizumab: 28 Cetuximab: 11 G12C inhibitor: 1
**Metastatic site at the initiation of the study**	Liver: 16 Lung: 16 Peritoneum: 5 Ovary: 3

**Table:** 

Primary assessment method	
Number of patients screened	34
Number of patients enrolled	28
Number of patients evaluable for toxicity	28
Number of patients evaluated for efficacy	24
Evaluation Method	RECIST 1.1
Response assessment, CR	0 (0%)
Response assessment, PR	2 (8.3%)
Response assessment, SD	18 (75%)
Response assessment, PD	4 (16.7%)
Median duration assessments, PFS	4.9 months (95% CI, 4.2-5.6)
Median duration assessments, OS	15.4 months (95% CI, 11.1-19.7)

## Outcome notes

### Patients

From March 1, 2022 to December 1, 2023, 34 patients were screened for eligibility. Twenty eight patients were enrolled to receive regorafenib plus TAS-102, and 24 could be assessed for efficacy (Figure 1). At the cutoff date of February 29, 2024, treatment was ongoing in 2 (7.1%) patients. 2 patients discontinued treatment for COVID-19 infection. The median number of treatment cycles was 4 (range: 1-12). 17 (60.7%) patients started regorafenib at a daily dose of 120 mg. 7 (25.0%) patients initiated at 80mg and then escalated to 120 mg daily after 1 week. 4 (14.3%) patients maintained at 80mg from the very beginning without subsequent escalation. Of note, 15 (53.6%) patients were administered bevacizumab as part of both first- and second-line treatments consecutively.

### Efficacy

**Figure 2.**
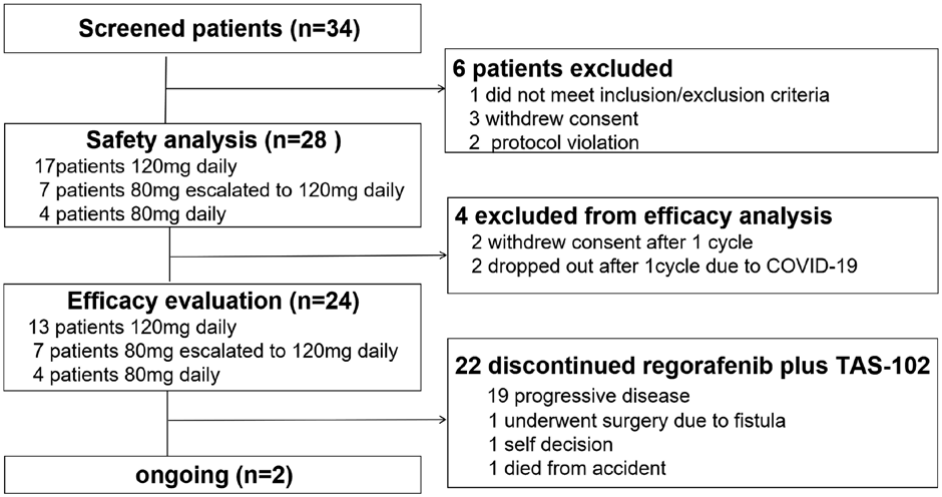
Progression free survival (A) and overall survival (B) in the 24 evaluable.

**Figure 3.**
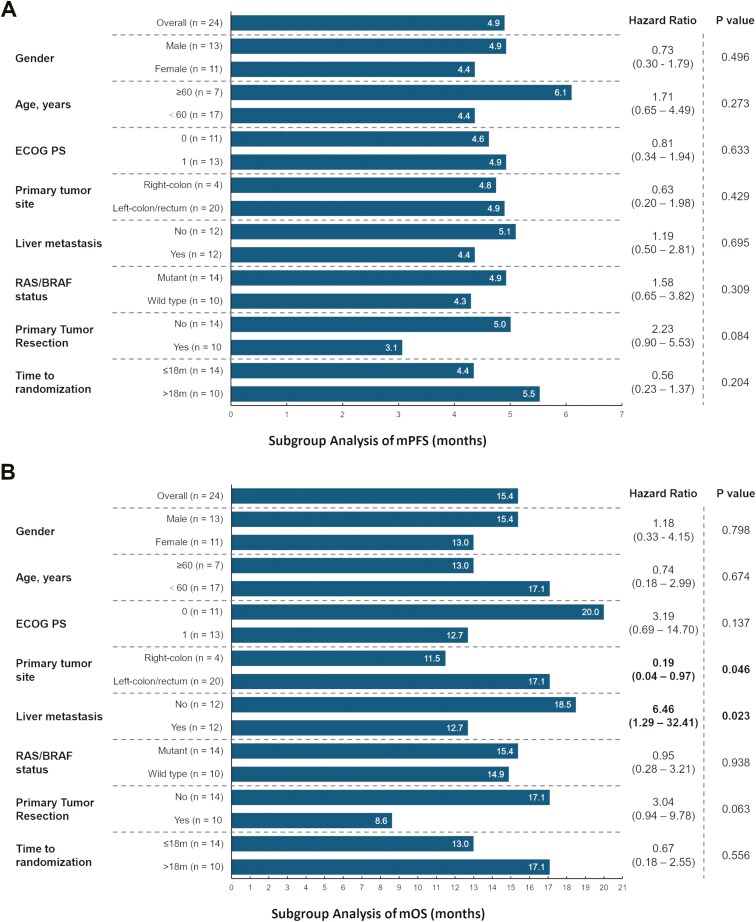
Exploratory subgroup analysis of progression free survival (A) and overall survival (B).

At the data cutoff, 25 (89.3%) patients in the intention-to-treat (ITT) group had progressed and 16 (57.1%) died. The median PFS was 4.9 months (95% CI, 4.2-5.6) in the 24 evaluable patients (Figure 2). Exploratory subgroup analyses indicated the PFS benefit in all subgroups (Figure 3A). No significant difference was seen in patients with or without liver metastasis (4.4 months vs 5.1 months, *P* = .70), as well as patients with RAS/BRAF mutations versus wild type counterparts (4.9 months vs 4.3 months, *P* = .30). Besides, the PFS seemed to favor those who were beyond 18 months from diagnosis of mCRC (5.5 months).

The median OS was 15.4 months (95% CI, 11.1-19.7, Figures 2 and 3B). Those who had a performance status score of 0 and beyond 18 months from diagnosis of mCRC had a favorable prognosis, with OS of 20.0 months and 17.1 months, respectively. Liver metastasis and right-sided location were associated with worse survival (Figure 3B).

**Figure 4.**
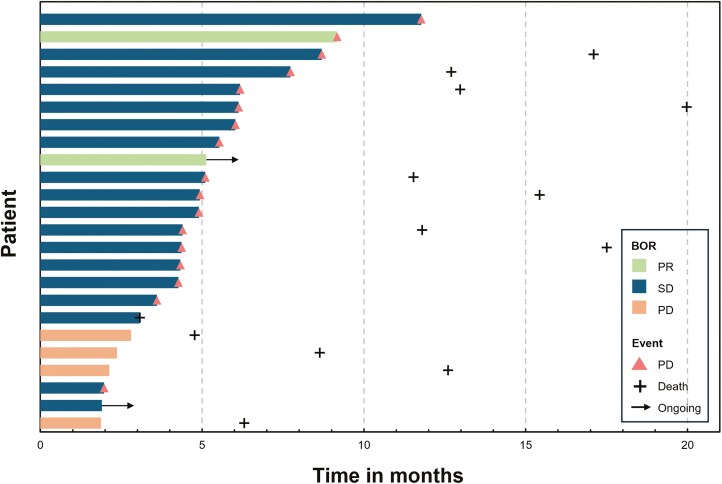
Swimmer plot showing the treatment efficacy, duration of disease control and overall survival.

As shown in the swimmer plot (Figure 4), 2 (8.3%) achieved PR and 18 (75.0%) had SD, 4 (16.7%) PD, with the ORR of 8.3% (95%CI, 2.3%-25.8%) and DCR of 83.3% (95%CI, 64.1%-93.3%). The two responders appeared in patients with lung metastasis. In the subgroup of liver metastasis, the ORR and DCR were 0.0% and 83.3%, respectively, which was consistent with the full population. 4 patients developed tumor progression at the first evaluation, of whom 2 patients had COVID-19 infection during the first or second cycle of treatment.

### Safety

All patients experienced at least one adverse event (Table 1), and the commonest treatment-related adverse effects (TRAE) of any grade included hypertension (67.9%), hand-foot skin reaction (60.7%), fatigue (50.0%), hoarseness (35.7%), reduced appetite (28.6%), leukopenia (32.1%), thrombocytopenia (21.4%), proteinuria (14.3%) and increased alanine aminotransferase (17.9%). Grade 3 or 4 TRAEs were reported in 21.4% of patients. The commonest grade 3 or higher adverse events included hypertension (7.1%), neutropenia (7.1%), thrombocytopenia (3.6%), and hoarseness (3.6%). One patient permanently discontinued study treatment because of a duodenal fistula that was considered to be related to regorafenib and underwent operation. No treatment-related deaths occurred.

**Table 1:** Treatment-related adverse events in the safety population (28 patients)

Any events	Regorafenib plus FTD–TPI (*n* = 28)				
	Any grade (%)	Grade 1 (%)	Grade 2 (%)	Grade 3 (%)	Grade 4 (%)
ALL	28 (100.0)	5 (17.9)	17 (60.7)	5 (17.9)	1 (3.6)
Hematological toxicity					
Leucopenia	9 (32.1)	6 (21.4)	2 (7.1)	1 (3.6)	0
Anemia	7 (25.0)	4 (14.3)	3 (10.7)	0	0
Thrombocytopenia	6 (21.4)	3 (10.7)	2 (7.1)	0	1 (3.6)
Neutropenia	5 (17.9)	3 (10.7)	0	2 (7.1)	0
Lymphopenia	2 (7.1)	2 (7.1)	0	0	0
Non-hematological toxicity					
Hypertension	19 (67.9)	7 (25.0)	10 (35.7)	2 (7.1)	0
Hand–foot skin reaction	17 (60.7)	11(39.3)	6 (21.4)	0	0
Fatigue	14(50.0)	9 (32.1)	5 (17.9)	0	0
Hoarseness	10 (35.7)	8 (28.6)	1 (3.6)	1 (3.6)	0
Decreased appetite	8(28.6)	6 (21.4)	2 (7.1)	0	0
Nausea	7 (25.0)	5 (17.9)	2 (7.1)	0	0
Diarrhea	5 (17.9)	3 (10.7)	2 (7.1)	0	0
Hyperbilirubinemia	5 (17.9)	4 (14.3)	1 (3.6)	0	0
Aminotransferase increased	5 (17.9)	3 (10.7)	2 (7.1)	0	0
Vomiting	4 (14.3)	2 (7.1)	2 (7.1)	0	0
Proteinuria	4 (14.3)	3 (10.7)	1 (3.6)	0	0
Hyperlipidemia	4 (14.3)	4 (14.3)	0	0	0
Hypoalbuminemia	3 (10.7)	1 (3.6)	2 (7.1)	0	0
Constipation	2 (7.1)	2 (7.1)	0	0	0
Abdominal pain	2 (7.1)	1 (3.6)	1 (3.6)	0	0
Oral mucositis	2 (7.1)	1 (3.6)	1 (3.6)	0	0
Rash	2 (7.1)	1 (3.6)	1 (3.6)	0	0
Hypokalemia	2 (7.1)	2 (7.1)	0	0	0
Headache	1 (3.6)	1 (3.6)	0	0	0
Alopecia	1 (3.6)	1 (3.6)	0	0	0
Duodenal fistula	1 (3.6)	0	0	1 (3.6)	0
Hyponatremia	1 (3.6)	1 (3.6)	0	0	0

Compared with the toxicity profile of bevacizumab plus TAS-102 in the SUNLIGHT study (Supplementary Figure S1), regorafenib plus biweekly TAS-102 had less grade 3 or higher adverse effects (21.4% vs 72.4%), which mostly resulted from the decreased incidence of hematological adverse effects. However, patients administered with regorafenib plus TAS-102 had more hypertension (67.9% vs 10.2%), fatigue (50.0% vs 21.5%), and hand-foot skin reaction (60.7% vs less than 10%) of any grade. 7 (25.0%) patients had regorafenib dose reduction (from 120 mg to 80 mg), and dose delays of more than 2 weeks occurred in 7 patients (25.0%). All dose reductions were because of non-hematological toxicities, namely hand–foot syndrome (*n* = 3), hypertension (*n* = 3) and proteinuria (*n* = 1). While for TAS-102, 7 patients experienced dose delay and 2 patients had dose reduction from 30 mg/m^2^ to 25 mg/m^2^ because of neutropenia.

### Subsequent treatment

Twenty (83.3%) of the 24 evaluable patients received subsequent treatment after progression. Fruquintinib alone or plus anti-PD-1 agents were administered in 8 patients. Three patients received bevacizumab plus chemotherapy regimens. Two patients received hepatic arterial infusion chemotherapy (HAIC), 1 patient was administered traditional Chinese medicine, and 1 underwent palliative radiotherapy.

### Assessment, analysis, and discussion

**Table:** 

Completion:	Study completed
**Investigator’s assessment:**	Active and should be pursued further

This trial studying the combination of regorafenib and biweekly TAS-102 offers a new avenue for the treatment of refractory mCRC. This approach provided promising benefits in terms of DCR, PFS, and OS. Of note, 53.6% of patients were treated with bevacizumab in both first- and second-line treatments in this study, in contrast to 31.6% in SUNLIGHT.^14^ The encouraging PFS (4.9 months) and OS (15.4 months) in this study underscored the efficacy of regorafenib plus TAS-102 to overcome resistance to prior bevacizumab treatment.

Additionally, this approach demonstrated a manageable safety profile, corroborating the TAS CC4 study^17^ and notably less adverse effects than a standard 4-week TAS-102 schedule.^13^ A critical aspect of this study was the determination of the optimal dosage for the regorafenib plus TAS-102 to balance efficacy with safety and enable patients to maintain salvage therapy as long as possible in the context of limited options. While the phase I REMETY study suggested a RP2D dose of TAS-102 at 25 mg/m^2^ bid, and regorafenib at 120mg on a standard 4-week schedule, the dosage was not deemed optimal due to manageable dose-limiting toxicities (DLTs) and a disease control rate of only 33.3% at this dose level.^18^

Regarding regorafenib, dose reductions with the initial 160 mg dose occurred in 67% and 71% of patients in the CORRECT and CONCUR trials, respectively.^3,4^ A tailored approach proved beneficial, starting at lower doses (80 or 120 mg/day) and escalating as tolerated,^19^ with more patients completing more cycles of therapy. The REARRANGE study further corroborated the benefits of reduced initial dosing of regorafenib.^20^ A maintenance dose of regorafenib not exceeding 120 mg for lighter patients (≤60kg) was reported to be effective and safe.^21^ When used in combination, the optimal dose of regorafenib remains unclear, which has been examined in several studies.^22,23^ The lower dose of regorafenib (80 mg in nearly 90% of patients) plus chemotherapy yielded a significant survival advantage without substantially increasing side effects.^24^ For TAS-102, this study opted for a biweekly administration to decrease unnecessary dose reductions and maintain effectiveness.^17,25^ Of note, the phase I REMETY study lacked a 30mg/m^2^ bid level of TAS-102,^16^ and pharmacokinetic data supported this dose level since Cmax and AUC_0-10_ between the doses of 60 and 70 mg/m^2^/day were comparable.^18^

Thus, the carefully adjusted dose and schedule of two drugs might contribute to the encouraging safety profile in this study. Other contributing factors, such as meticulous monitoring, improved adverse event management strategies, and patient education, should not be overlooked for the safety improvement. The high rate of hypertension (67.9%) might be related to a large proportion of patients (46.4%) with a baseline medical history of hypertension.

While the combination of TAS-102 with bevacizumab has emerged as a compelling option,^13^ immune checkpoint inhibitors have broadened the therapeutic landscape in MSS-refractory mCRC. The presence of liver metastases remains a challenge in immunotherapy.^26^ By contrast, the combination of TAS-102 with regorafenib in this study or bevacizumab in a previous trial^27^ achieved substantial DCR and PFS, irrespective of liver metastasis. Therefore, anti-angiogenic agents plus TAS-102 would be a preferable option in the setting of liver metastasis.

This study, while promising, had limitations. The non-randomized design and small sample size warrant caution in data interpretation. The absence of life qualities assessments also points to areas where further research is needed. The simplicity and oral administration of this regimen could be advantageous for patients and healthcare providers alike, but larger, randomized controlled trials are warranted to confirm the above findings and to refine patient selection criteria. Since only 2 patients experienced TAS-102 dose reduction, maybe the biweekly schedule of 35mg/m^2^ bid could be explored in the future when in combination with regorafenib.

In summary, the REGTAS study suggests that a combination of regorafenib and biweekly TAS-102 is a feasible, well-tolerated, and effective treatment option for refractory mCRC.

## Supplementary Material

oyaf129_suppl_Supplementary_Figure_1

## Data Availability

The data underlying this article are available in the article and in its online supplementary material.
