# Supplementary material for: Efficacy and safety of regorafenib plus biweekly trifluridine/tipiracil for refractory metastatic colorectal cancer: a multicenter single-arm phase II trial
Source: Oncologist. 2025 Jun 17;30(6):oyaf129. doi: 10.1093/oncolo/oyaf129 (PMC12200236; doi:10.1093/oncolo/oyaf129)
Supplement: oyaf129_suppl_Supplementary_Figure_1 [file oyaf129_suppl_supplementary_figure_1.docx]

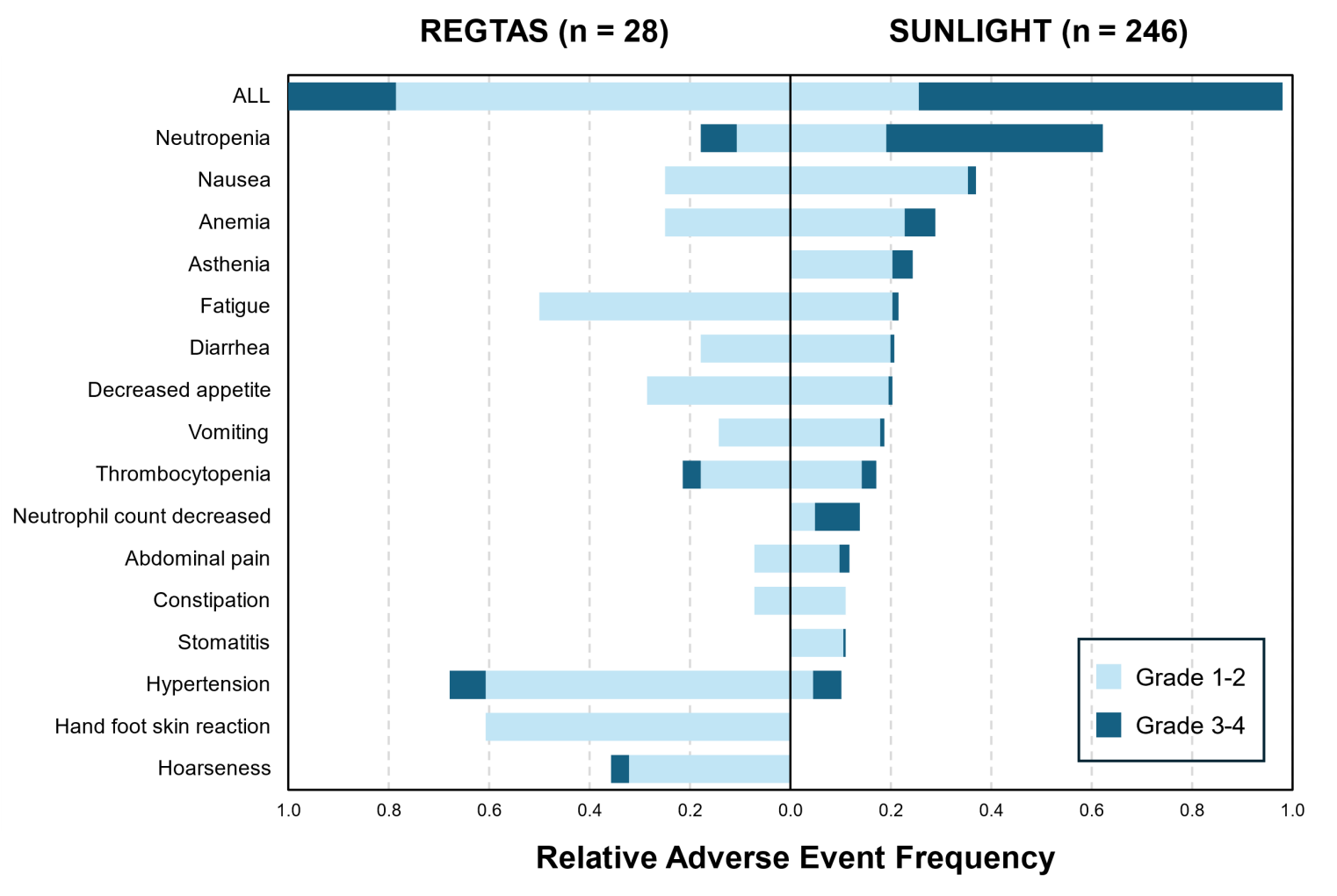


Supplementary Figure 1. Indirect comparison of toxicity spectrum of bevacizumab plus TAS-102 in SUNLIGHT13 study and regorafenib plus TAS-102 in this study.
